# Evaluation of Ventricular Remodeling and Prognosis in Patients with Aortic Stenosis Who Underwent Surgical or Percutaneous Transcatheter Aortic Valve Replacement

**DOI:** 10.21470/1678-9741-2021-0175

**Published:** 2022

**Authors:** Rodrigo de Moura Joaquim, Tiago Ghislandi Nuernberg, Tammuz Fattah, Roberto Leo da Silva

**Affiliations:** 1 Interventional Cardiology Service, Instituto de Cardiologia de Santa Catarina, São José, Santa Catarina, Brazil.; 2 Interventional Cardiology Service, Hospital Universitário Professor Polydoro Ernani de São Thiago, Florianópolis, Santa Catarina, Brazil.

**Keywords:** Aortic Valve, Aortic Valve Stenosis, Heart Valve Diseases, Echocardiography, Usage Remodeling, Ventricular Remodeling, Surgical Instruments

## Abstract

**Introduction:**

Aortic stenosis is the most common heart valve disease in the world, and patients that present with symptoms have a high mortality rate. Aortic valve replacement has the objective of promote left ventricular remodeling, reduce symptoms, and increase overall survival. The objective of this study is to evaluate reverse remodeling of the left ventricle in patients with severe and symptomatic aortic stenosis who underwent surgical or percutaneous transcatheter aortic valve replacement.

**Methods:**

This is a longitudinal, prospective, non-concurrent, non-randomized unicentric study with patients who underwent aortic valve replacement. Echocardiogram was performed before and after replacement procedure to evaluate several remodeling indexes.

**Results:**

Of 91 patients, 77 (84.6%) underwent surgical aortic valve replacement, and 14 (15.4%) underwent percutaneous transcatheter aortic valve replacement. Mean age was 68,96±11,98 years, and most patients were male. Remodeling evaluation revealed that patients who decreased left ventricular index mass (53% vs. 38.9%; P=0,019) and those who reduced the mass/volume ratio (30.4% vs. 68.9%; P<0,001) presented with positive left ventricular remodeling. No endpoint difference was found in those with positive remodeling.

**Conclusion:**

Regarding the left ventricular remodeling in patients with severe and symptomatic aortic valve stenosis who underwent percutaneous transcatheter or surgical valve replacement, there is a positive increment in remodeling, however it remains in concentric hypertrophic shape. Implication of these findings remains uncertain and to be studied in large dedicated trials with clinical endpoints.

**Table:** Abbreviations, Acronyms & Symbols

AS	= Aortic stenosis	LVMI	= Left ventricular mass index
CAD	= Coronary artery disease	LVPW	= Left ventricular posterior wall
CI	= Confidence interval	MI	= Myocardial infarction
EF	= Ejection fraction	OR	= Odds ratio
ICU	= Intensive care unit	PAP	= Pulmonary artery pressure
LV	= Left ventricular	PASP	= Pulmonary artery systolic pressure
LVDVI	= Left ventricular diastolic volume index	RWT	= Relative wall thickness
LVEDD	= Left ventricular end-diastolic diameter	SAVR	= Surgical aortic valve replacement
LVEDV	= Left ventricular end-diastolic volume	TAVR	= Transcatheter aortic valve replacement
LVEF	= Left ventricular ejection fraction		

## INTRODUCTION

Aortic stenosis (AS) is the most prevalent heart valve disease in the world, and symptomatic patients have high rates of morbidity and mortality^[1-3]^. As stenosis progresses, there is an increase in the afterload; increased left ventricular (LV) pressure and adaptive hypertrophy to maintain ventricular wall stress can cause an inappropriate hypertrophic response, leading to fibrosis, dilation, and loss of ventricular function and resulting in symptoms, heart failure, arrhythmia, and death^[3-7]^. Reestablishing the valve function with an increase in the effective orifice area, reduction of transvalvular gradient, and reversal of hypertrophy results in improvements of symptoms and increased survival^[3-5]^.

The definitive treatment for AS is valve replacement, and the gold standard procedure is surgical aortic valve replacement (SARV), but it has been accompanied, in the last decade, by percutaneous transcatheter aortic valve replacement (TAVR) in low, intermediate, and high-risk surgical cases, with overlapping clinical results in both therapeutic methods^[8-13]^. Treatment is usually indicated according to stenosis severity, symptoms, and ejection fraction (EF)^[1-2]^.

Reverse remodeling is a phenomenon that occurs in patients with AS after valve replacement, and it is characterized by enhance in hypertrophy pattern followed by ventricular mass regression and increase in ventricular function, evaluated through echocardiogram or magnetic resonance^[3,4,14-16]^. Greatest reduction usually occurs within the first six months, but it persists improving until up to two years after valve replacement, accompanied by reduction in the LV mass/volume ratio, reduction in cavitary volumes, and enhance in diastolic filling and global heart function^[5-7,15-17]^.

The aim of this study is to perform an echocardiographic evaluation of aortic valve replacement effects in LV remodeling in patients with severe symptomatic AS who underwent SARV or TARV.

## METHODS

A longitudinal, prospective, non-concurrent, non-randomized unicentric trial was performed in patients with AS who underwent SAVR or TAVR from January 2014 to December 2018 in a large tertiary cardiology center. Data collection was carried out mostly through review of electronic medical records, with complement of physical records, when necessary, without contact with patients or interference in their treatment, therefore informed consent was dispensed.

Research was approved by local institution’s ethics and humans research independent committee through Plataforma Brasil, under protocol nº 3.593.226.

### Population and Data Collection

Patients > 18 years of age, with severe and symptomatic AS, and submitted to SAVR or percutaneous TAVR were included. Severe AS was defined as a valvar area ≤ 1 cm^2^ or indexed valve area ≤ 0.6 cm^2^/m^2^, mean aortic valve gradient ≥ 40 mmHg, and maximum jet velocity ≥ 4 m/s; patients with low flow/low gradient AS with aortic valve area ≤ 1 cm^2^ with LV ejection fraction (LVEF) < 50%, and mean aortic valve gradient ≤ 40 mmHg, but who presented myocardial reserve, were also considered^[1]^. Symptoms were chest pain, syncope or dyspnea, and functional class ≥ 2 secondary to valvar disease. Patients with pacemakers, cardiac resynchronizers or implantable defibrillators, hypertrophic cardiomyopathy with or without outflow tract obstruction, myocardial infiltrative disease, predominance of aortic regurgitation, presence of infectious endocarditis or metallic or biological prosthesis in the aortic position, major LV dysfunction (EF < 20%), deaths in the perioperative period, and those who did not have echocardiogram data before or after valve replacement were excluded from the present study.

Data collection was related to characteristics and clinical evaluation, characteristics of valve replacement procedures, length of hospital stay, and complications that occurred both in the perioperative period and in clinical follow-up. The baseline echocardiogram used was the one performed closest to the valve replacement, no later than six months before the procedure. Follow-up echocardiogram was performed preferably between six and 24 months after the procedure. When the patient had more than one echocardiogram, the examination performed closer to 12 months of valve replacement was chosen for analysis. Echocardiographic data evaluated and collected from both echocardiograms were LV end-diastolic diameter (LVEDD) and diameters of other cavities, thicknesses of septal and posterior walls, LVEF, LV mass index (LVMI), and LV diastolic volume index (LVDVI), after their calculations, and relative wall thickness (RWT) (calculated with the formula 2 × posterior wall thickness/end-diastolic diameter)^[5]^.

### Outcomes

The primary objective of the study was to evaluate the impact of aortic valve replacement on reverse LV remodeling in patients with AS, evaluating LVMI, LVDVI, type of ventricular hypertrophy, mass/volume ratio, RWT, and LVEF before and after the procedure. As secondary objective, the characteristics associated with improvement in the remodeling variables evaluated in the primary outcome were analyzed. Variables were then associated with a defined composite outcome of death by all causes, need for a new pacemaker implantation, valve prosthesis dysfunction, considered stenosis, significant valve insufficiency or paravalvular leak, and need for valve replacement in the follow-up.

### Statistical Analysis

Statistical analysis was performed with the IBM Corp. Released 2013, IBM SPSS Statistics for Windows, version 22.0, Armonk, NY: IBM Corp. program. Continuous variables were expressed as means and a standard deviation and, when necessary, they were compared using the Student’s *t*-test or the Mann-Whitney U test, when the continuous data did not have a normal distribution, for paired samples. Categorical variables were described as frequencies and percentages and were compared using the chi-squared test or Fisher’s exact test. A multiple regression model was used to evaluate those variables that were associated with the increase in remodeling and the events in the follow-up. Variables that presented a modestly close relationship with *P*<0.3 were included in the multiple regression model. The significance level used in all evaluations was a two-sided *P*-value of 0.05. The continuous variables were categorized according to their means, when appropriate for their association.

## RESULTS

**Fig. 1:**
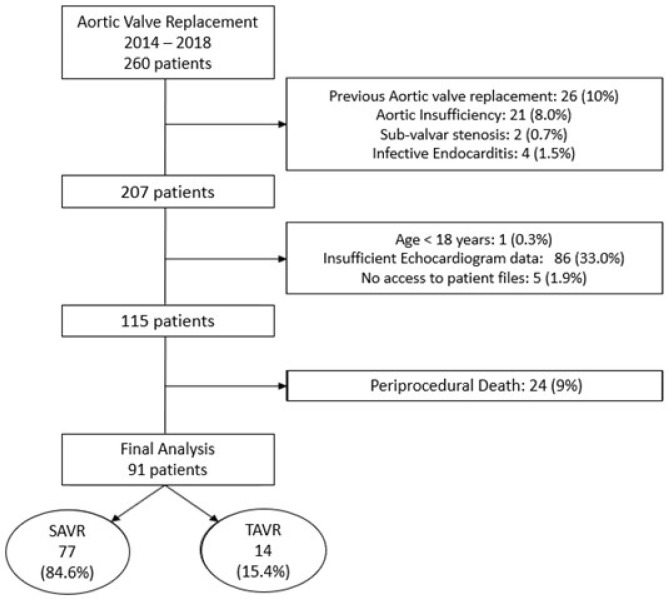
Patient inclusion algorithm. SAVR=surgical aortic valve replacement; TAVR=transcatheter aortic valve replacement

From January 2014 to December 2018, 260 patients with symptomatic AS underwent valve replacement in our institution. Of these, 169 were excluded because they did not meet inclusion criteria. Figure 1 details the exclusion reasons and final population. A total of 91 patients were included for final analysis, of which 77 (84.6%) underwent SAVR, 24 (31%) with mechanical prosthesis and 53 (69%) with biological prosthesis, and 14 (15.4%) underwent TAVR. Mean age was 68.96 (±11.98) years, and 65% of the patients were male. The main baseline characteristics are summarized in Table 1. Four (4.4%) patients had history of previous stroke, and four (4.4%) of chronic obstructive pulmonary disease. Thirteen (14.4%) patients had previous coronary revascularization, 11 (12.2%) percutaneous coronary intervention, and two (2.2%) myocardial revascularization surgery.

**Table 1:** Overall population characteristics.

Variable	n (%)
Sex, female	32 (25.2%)
Hypertension	59 (64.8%)
Diabetes	24 (26.4%)
Dyslipidemia	36 (39.6%)
Smoker	10 (11%)
Chronic renal disease	26 (25.8%)
CAD	44 (48.4%)
Previous MI	15 (16.5%)

CAD=coronary artery disease; MI=myocardial infarction

### Hospitalization and Perioperative Characteristics

Aortic valve replacement was combined with other procedures in 23 (25.3%) cases — 15 cases (16.5%) with coronary revascularization. During hospitalization in the postoperative period, 60 (65.9%) patients had an event. The most common events were bleeding and occurrence of atrial fibrillation in 12 (13.1%) and 24 (26.3%) patients, respectively. There were also three (3.2%) cases of acute myocardial infarction, six (6.5%) patients evolved with acute renal injury, six (6.5%) with total atrial-ventricular block, and four (4.3%) with need for a definitive pacemaker implantation. Postoperatively, mean time in intensive care unit (ICU) was 4.56±4.43 days, and mean length of hospital stay after valve replacement was 14.31±13.59 days, including the mean ICU time. Twenty-four (26.4%) patients died in the postoperative period and were not included in the final analysis (Figure 1).

### Ventricular Remodeling

**Fig. 2:**
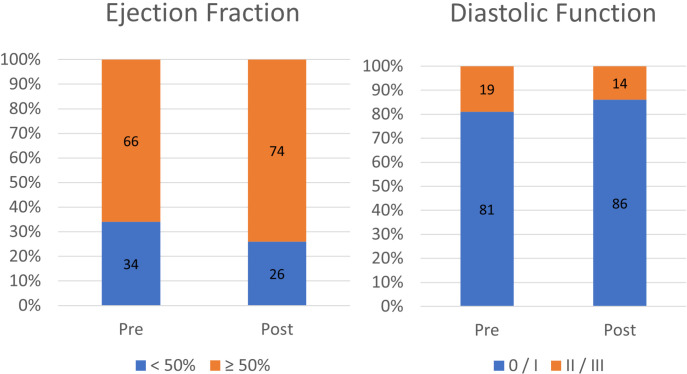
Left ventricular function pre- and post-aortic valve replacement.

Echocardiographic data at baseline and after aortic valve replacement are presented in Table 2. Mean follow-up exam was performed within 14.64±12.96 months. A significant reduction in LVEDD and thickness of the interventricular septum and posterior LV wall was observed, also resulting in the reduction of LVMI, LVDVI, RWT, and mass/volume ratio in the follow-up echocardiogram. LVEF showed an increase in relation to baseline but without statistical significance. Figure 2 shows the increase in EF and diastolic function after valve replacement.

**Table 2:** Echocardiography data at baseline and after aortic valve replacement.

	Baseline Echocardiogram	Follow-up Echocardiogram	*P*-value*
Aorta (cm)	3.434 (± 0.551)	3.293 (± 0.575)	-
Left atrium (cm)	4.241 (± 0.842)	4.308 (± 0.727)	-
LVEDD (ml)	5.088 (± 0.818)	4829 (± 0.793)	0.004
Interventricular septum (cm)	1.403 (± 0.249)	1.208 (± 0.234)	< 0.001
LVPW (cm)	1.347 (± 0.258)	1.160 (± 0.249)	< 0.001
RWT (cm)	0.535 (± 0.125)	0.486 (± 0.126)	0.003
LVMI (g/m2)	179.372 (± 83.903)	124.801 (± 40.700)	< 0.001
LVDVI (ml/m2)	75.921 (± 37.517)	63.713 (± 27.691)	0.001
Mass/volume	2.577 (± 1.244)	2.175 (± 0.922)	0.014
LVEF (%)	0.560 (± 0.188)	0.588 (± 0.139)	0.087
PAP (mmHg)	40.110 (± 14.868)	33.320 (± 11.009)	-
Maximum gradient	76.290 (± 20.707)	24.900 (± 12.408)	-
Medium gradient	48.270 (± 13.643)	15.840 (± 9.575)	-

LVDVI=left ventricular diastolic volume index; LVEDD=left ventricular end-diastolic diameter; LVEF=left ventricular ejection fraction; LVMI=left ventricular mass index; LVPW=left ventricular posterior wall; PAP=pulmonary artery pressure; RWT=relative wall thickness

* Student’s *t*-test or Wilcoxon-Mann-Whitney U test

Seventeen (19%) patients had moderate to important aortic regurgitation associated with stenosis before valve replacement. After the procedure, this number was reduced to three (4%). A reduction in the maximum and mean valve gradients was also observed, in addition to a slight reduction in pulmonary artery systolic pressure (PASP). As not all patients had measurements of PASP and valve gradients on the follow-up echocardiogram, no additional analyses were performed with these variables.

**Fig. 3:**
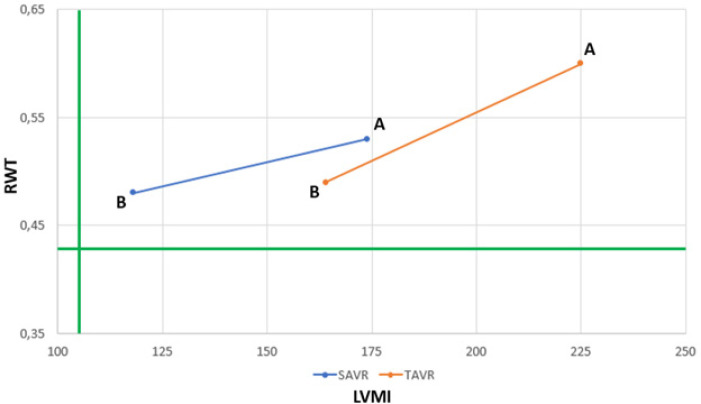
Left ventricular geometry representation. Normal geometry: RWT < 0,43 and LVMI < 105 g/cm2. Concentric hypertrophy: RWT > 0,43 and LVMI > 105 g/cm^2^. A=baseline; B=follow-up; RWT=relative wall thickness; LVMI=left ventricular mass index; SAVR=surgical aortic valve replacement; TAVR=transcatheter aortic valve replacement

Despite the improvement in various remodeling indexes observed after the procedure, ventricular geometry remained similar, with a concentric hypertrophic LV shape, either after SAVR or TAVR (Figure 3).

### Factors Associated with Ventricular Remodeling

The remodeling indexes, including LVMI, LVDVI, RWT, and mass/volume ratio, were dichotomized according to the difference of their means, and the LVEF was evaluated according to ≥ 10% increase of their values after valve replacement, in order to evaluate factors associated with the improvement of these indexes.

The factors related to the greatest reduction in LVMI were male sex (50.8% *vs*. 28.1%; *P*=0.047), patients with reduced LVEF (< 50%; 64.5% *vs*. 32.1%; *P*=0.003), LVMI values (75.7% *vs.* 20.3%; *P*<0.001), and LV end-diastolic volume (LVEDV) (64.1% *vs.* 26.9%; *P*=0.001) higher than the average on baseline echocardiogram. After logistic regression model that also involved age and smoking, the baseline LVMI remained the only variable associated with improvement in LVMI (*P*=0.001; odds ratio [OR]: 1,033; 95% confidence interval [CI] 1.016-1.050). Only patients with LVDVI ≥ 75 mL/m2cs at baseline echocardiogram presented a significant reduction in LVDVI after valve replacement (50.0% vs. 20.5%; *P*=0.004), as higher the initial LVDVI, greater the regression of the same index was. This association was lost in multivariate analysis (*P*=0.370).

Regarding RWT, not presenting a previous myocardial infarction (53.9% *vs*. 13.3%; *P*=0.04) and a higher RWT at baseline echocardiogram (57.7% *vs*. 10.5%; *P*<0.001) were associated with its regression. In a multivariate regression model, which included smoking, LVEDV, and LVEF, the RWT of the baseline echocardiogram remained associated with enhanced ventricular remodeling (*P*=0.01; OR: 2472.3; 95% CI 6.3 - 960543.5). Three factors were associated with an increase of at least 10% in LVEF after valve replacement, high baseline LVEDV (50.0% *vs*. 16.0%; *P*=0.001), low baseline RWT (44.4% *vs.* 21.7%; *P*=0.001), and reduced baseline LVEF (< 50%; 64.5% *vs.* 12.3%; *P*<0.001). In this evaluation, patients with eccentric hypertrophic pattern had a higher chance of increasing LVEF. This association was lost in multivariate analysis. No variable analyzed was associated with improvement of the mass/volume ratio.

### Compound Secondary Outcome

The mean follow-up time was 2.6±1.6 years, and 12 (13.2%) patients had an event in the composite outcome. The occurrence of perioperative events (25.6% *vs*. 2.1%; *P*=0.001) and the non-increase of at least 10% in LVEF after valve replacement (18% *vs*. 0.0%; *P*=0.016) were the only two factors associated with the events that occurred during the clinical follow-up period. After adjustments in multivariate regression analysis, no association with the events was detected.

## DISCUSSION

Our study reached its primary objective and demonstrated that after valve replacement in symptomatic patients with AS, there is an enhance in LV remodeling rates through echocardiographic analysis, with reduction of LVMI, LVDVI, RWT, and mass/volume ratio. The higher these indices are preoperatively, more pronounced the reduction and benefit are in remodeling, but ventricular geometry remained with a concentric hypertrophic pattern despite the clear regression, both in patients treated with SARV and TAVR. Despite an improvement in remodeling rates was evaluated, there was no relation with occurrence of adverse events in a medium-term follow-up. It is noteworthy that patients who presented an increase of at least 10% in LVEF did not present events in the follow-up.

With barrier elimination and afterload reduction, patients submitted to aortic valve replacement present various degrees of reduction in ventricular mass, with an increase in volumes and improvement of LV diastolic and systolic functions^[3,4,14]^. La Manna et al.^[15]^ evaluated 27 patients submitted to TAVR with magnetic resonance imaging at six months and identified that, even in this short period, LVMI regression occurs (84.5±25.2 g/m^2^ *vs*. 69.4±18.4 g/m^2^; *P*<0.001), observed in patients with or without myocardial fibrosis, but with no difference in LVDVI, LVEF, and systolic volume. In this analysis, the authors indicated a reverse remodeling with reduction of the mass/volume ratio (*P*=0.001) at the expense of LVMI, but no factor was a predictor of reverse remodeling in multivariate analysis. The reduction of LVMI starts early and seems to persist until at least four years after aortic valve replacement, but its effect on remodeling and final ventricular geometry is still uncertain^[7,16]^. In 50 high-risk patients, with a mean age of 77 years, with symptomatic AS, Fairbairn et al.^[6]^ also demonstrated a reduction in LVMI and LV systolic volume with both SAVR and TAVR. In this study, only patients submitted to SARV^[25]^ presented a significant reduction in LVDVI (92±19 ml/m^2^ *vs*. 74±12 ml/m^2^; *P*<0.001) and baseline LV volumes were predictors of reverse remodeling (*P*<0.001). In our study, the factors associated with increased remodeling after multivariate analysis were LVMI and LVDVI. We found a slight and non-significant increase in LVEF after valve replacement. This increase is observed in several studies and, in general, is important after procedure, particularly in patients with previous reduced LVEF^[5,6,15]^. Age, gender, presence of fibrosis, and myocardial reserve are usually factors related to the improvement in LVEF, despite none was found in our analysis^[3,4]^.

In an echocardiographic sub-study of The Nordic Aortic Valve Intervention (or NOTION), Ngo et al.^[5]^ compared patients undergoing SAVR and TAVR at three and 12 months. They found similar reduction in RWT in both groups and a more marked reduction in LVMI in patients undergoing SAVR (17.5% *vs*. 7.2%; *P*<0.001). Thus, ventricular geometry, with a concentric hypertrophic pattern in both groups before the procedure, changed only in patients undergoing SAVR, moving to a concentric remodeling pattern. This was probably observed by the increase in LVEDV of patients undergoing TAVR due to factors such as aortic regurgitation and the need for pacemaker implantation. Lamb et al.^[16]^ have already demonstrated in the past that early reduction of LVMI is accompanied by reduction of mass/volume ratio improving ventricular remodeling and diastolic filling of those patients. Despite the increase in remodeling rates, our patients remained in a spectrum of LV concentric hypertrophy (Figure 3), a fact demonstrated both in patients who underwent SARV and TAVR, which can be explained in part by the baseline indexes being higher in our population, possibly by delayed treatment, with no adequate global change. SARV and TAVR seem to promote an increase in remodeling despite small differences in some indexes found in the studies^[5,6]^.

We did not demonstrate the impact of different remodeling indexes on patient prognosis. An analysis of 4,280 patients identified that the presence of important LV hypertrophy before valve replacement was associated with an increase of 16% in mortality and 34% in the compound outcome of mortality and the need for new hospitalization at five-year follow-up. Mild or moderate hypertrophy was not associated with outcomes^[18]^. In an evaluation, with a five-year follow-up, of 1,434 patients who were alive one year after TAVR, Chau et al.^[17]^ demonstrated an average reduction of 14% in LVMI, with reduced mortality and need for further hospitalization. For each 10% reduction of LVMI, there was a reduction of 5% in mortality. The presence of significant LV hypertrophy in one year (39% of patients) was also associated with all-cause mortality (adjusted risk ratio 1.71; 95% CI 1.20 - 2.44; *P*=0.003). On the other hand, a study with 31,199 patients from 422 centers of the Society of Thoracic Surgeons/American College of Cardiology Transcatheter Valve Therapy (or STS-ACC TVT) Registry, which evaluated patients undergoing TAVR, found no association between concentric or eccentric ventricular hypertrophy when a one-year death, myocardial infarction, stroke, or need for dialysis endpoint was evaluated^[19]^. Despite some contradictory data, the presence of greater ventricular hypertrophy before valve replacement and a less significant reduction of LVMI seems to be associated with a worse prognosis in these patients, indicating that an earlier intervention according to overall LV characteristics, which goes beyond LVEF, seems to be the way to improve prognosis in this population.

### Limitations

Our study has several limitations. Due to high rates of patient exclusion, accounting for approximately 2/3 of total procedures, most due to the absence of all necessary data, a small sample was submitted to final analysis. Patient-prosthesis mismatch and other valve hemodynamics measures were not performed and analyzed due to lack of echocardiogram data and may have impact on LV remodeling. The heterogeneity of the sample, which included patients undergoing SAVR with mechanical, biological, and TAVR prosthesis, may also have some influence on the results due to the different populations that usually receive each of these devices. The exclusion of patients with death in the perioperative period was necessary to be able to complete the follow-up and to have at least one echocardiographic examination in the follow-up, but this also causes a population of lower risk for future events, leading to a low incidence of outcomes in the medium-term follow-up. Finally, the non-complete return to normal LV geometry, remaining the pattern of concentric hypertrophy in the population mean, may indicate that patients were late submitted to valve replacement with worse remodeling and LV function, consequently.

## CONCLUSION

Our study concludes that in patients with severe and symptomatic AS, remodeling indexes including LVMI, LVDVI, RWT, and mass/volume ratio enhance after aortic valve replacement. This is more evident in patients with higher initial mass and volumes, did not result in changes of LV final geometry, and was not associated with reduction in population events in a medium-term follow-up.

**Table:** Authors' roles & responsibilities

RMJ	Substantial contributions to the design of the work; and the acquisition of data for the work; drafting the work; final approval of the version to be published
TGN	Substantial contributions to the design of the work; and the acquisition of data for the work; drafting the work; final approval of the version to be published
TF	Substantial contributions to the design of the work; and the acquisition, analysis, and interpretation of data for the work; final approval of the version to be published
RLS	Substantial contributions to the analysis and interpretation of data for the work; final approval of the version to be published
